# Voxel-Wise Linearity Analysis of Increments and Decrements in BOLD Responses in Human Visual Cortex Using a Contrast Adaptation Paradigm

**DOI:** 10.3389/fnhum.2021.541314

**Published:** 2021-08-31

**Authors:** Yun Lin, Xi Zhou, Yuji Naya, Justin L. Gardner, Pei Sun

**Affiliations:** ^1^Department of Psychology, School of Social Sciences, Tsinghua University, Beijing, China; ^2^School of Psychological and Cognitive Sciences, Peking University, Beijing, China; ^3^Department of Psychology, Stanford University, Stanford, CA, United States; ^4^Tsinghua Laboratory of Brain and Intelligence, Tsinghua University, Beijing, China; ^5^Laboratory for Cognitive Brain Mapping, RIKEN Center for Brain Sciences, Wako, Japan

**Keywords:** fMRI, linearity, BOLD signal decrement, visual cortex, contrast adaptation

## Abstract

The linearity of BOLD responses is a fundamental presumption in most analysis procedures for BOLD fMRI studies. Previous studies have examined the linearity of BOLD signal increments, but less is known about the linearity of BOLD signal decrements. The present study assessed the linearity of both BOLD signal increments and decrements in the human primary visual cortex using a contrast adaptation paradigm. Results showed that both BOLD signal increments and decrements kept linearity to long stimuli (e.g., 3 s, 6 s), yet, deviated from linearity to transient stimuli (e.g., 1 s). Furthermore, a voxel-wise analysis showed that the deviation patterns were different for BOLD signal increments and decrements: while the BOLD signal increments demonstrated a consistent overestimation pattern, the patterns for BOLD signal decrements varied from overestimation to underestimation. Our results suggested that corrections to deviations from linearity of transient responses should consider the different effects of BOLD signal increments and decrements.

## Introduction

Among the different types of brain imaging techniques, blood oxygen level dependent (BOLD) functional magnetic resonance imaging (fMRI) is known as a powerful and non-invasive technique for the detection of brain neural activities (Deyoe et al., 1994; Logothetis et al., 2001; Logothetis and Wandell, 2004). Relying on observing the underlying hemodynamic changes within the brain, this technique has been adopted to study and identify brain regions or networks that are associated with perception, attention, memory, and other mental processes, covering a wide range of research topics (Logothetis, 2008; Kim, 2011; Cortese et al., 2012; Peelle, 2014). With BOLD fMRI becoming one of the most common brain mapping tools since 1992 (Bandettini et al., 1992; Blamire et al., 1992; Kwong et al., 1992; Ogawa et al., 1992), different data analysis procedures have been developed to better study the observations that are obtained by this imaging technique, with most of them presuming a temporal linear relationship between the stimuli and BOLD responses (Friston et al., 1994a, b; Poline and Brett, 2012). Yet, this linear relationship can only be established when the BOLD response to the sum of stimuli equals the temporal linear summation of BOLD responses to each stimulus (Boynton et al., 1996; Birn et al., 2001). Particularly, if the linearity holds, and there is a long stimulus that can be divided into a shorter stimulus and its temporally shifted copies, then the BOLD response to the long stimulus should be approximated by the summation of the BOLD response to the shorter stimulus and its temporally shifted copies. This property is referred to as the superposition principle of linearity. The present study focuses on the investigation of the superposition property of BOLD signal decrements, shedding light on the data analysis procedures for the BOLD signal decrements.

Boynton et al. (1996) demonstrated the linearity of BOLD signal increments in an earlier study, showing that the BOLD signal increment to long visual stimulus could be predicted by the summation of the BOLD signal increment to shorter visual stimulus and its temporally shifted copy or copies (Boynton et al., 1996). While the linearity of BOLD signal increments to long stimuli has been confirmed (Birn et al., 2001; Soltysik et al., 2004), several studies reported non-linearity for BOLD signal increments to transient stimuli (Savoy et al., 1995; Konishi et al., 1996; Friston et al., 1998; Robson et al., 1998; Vazquez and Noll, 1998; Birn et al., 2001; Soltysik et al., 2004; Wager et al., 2005; Thompson et al., 2014; Alahmadi et al., 2017). Specifically, the predictions made by BOLD signal increments to transient stimuli tended to overestimate the BOLD signal increments to longer stimuli. Previous studies showed the overestimation pattern for the BOLD signal increments in the human primary visual, motor, and auditory cortices by using stimuli with durations less than 3, 7, and 10 s (Soltysik et al., 2004). The degree of overestimation increased as the stimulus duration decreased. For instance, studies revealed a slight overestimation tendency for BOLD signal increments to 3-s visual stimulus and an apparent overestimation pattern for BOLD signal increments to 1-s visual stimulus (Boynton et al., 1996; Vazquez and Noll, 1998; Birn et al., 2001; Soltysik et al., 2004).

In contrast to the various studies that tested the linearity of BOLD signal increments, the linearity of BOLD signal decrements has rarely been examined. While the BOLD signal increments are thought to reflect the localized neural activity (Deyoe et al., 1994; Logothetis et al., 2001; Heeger and Ress, 2002; Arthurs and Boniface, 2003), the underlying neural-physiological processes for BOLD signal decrements remain unclear (Birn and Bandettini, 2005; Tang et al., 2009). The linearity of BOLD signal decrements was analyzed in two previous studies which focused on the BOLD signal decrease in response to visual stimulus cessation. Results showed a pattern of underestimation for signal decrements to stimulus cessations that were 3-s or shorter while confirming the linearity for signal decrements to longer stimulus cessations (Birn and Bandettini, 2005; Gardner et al., 2005a; Tang et al., 2009). However, the BOLD signal decrements in these studies have been averaged out across all selected voxels, either for each participant or across participants, leaving the voxel-wise variations for the linearity of BOLD signal decrements an uncertain element. The deviations from linearity could have a huge impact on the robustness of related analysis procedures, yet the linearity has been presumed in the analysis procedures for most BOLD fMRI studies (including the studies for BOLD signal decrements). Moreover, while voxel-wise analysis has been commonly applied in many BOLD fMRI studies, relevant studies in observing the BOLD signal decrements are limited, making it necessary to investigate the linear properties of the BOLD signal decrements in a voxel-wise manner.

The present study assessed the voxel-wise linearity of both BOLD signal increments and decrements in the human primary visual cortex (V1) using a contrast adaptation paradigm, and a direct comparison between this linearity was made across BOLD response types. Participants were presented with an intermediate contrast level visual stimulus for visual contrast adaption. They were then introduced to increments and decrements of contrast level to elicit BOLD signal increments and decrements, respectively. The increments and decrements of contrast level were set to last for 1, 3, or 6 s, with reference to the stimulus durations that were frequently used in event-related BOLD fMRI studies (Huettel, 2012). Linearity was tested by calculating the similarity between linear predictions made by the BOLD responses to the short stimulus and the measured BOLD responses to the long stimulus in a voxel-wise manner. Results showed that both BOLD signal increments and decrements behaved in a more linear way for long stimuli (e.g., 3 s, 6 s) when compared with transient stimuli (e.g., 1 s). Moreover, while the BOLD signal increments demonstrated a consistent overestimation pattern, the patterns for BOLD signal decrements varied from overestimation to underestimation.

## Materials and Methods

### Participants

Twelve healthy right-handed participants (five males and seven females, aged between 19 and 29 years) with normal or corrected-to-normal vision were recruited in the present study. The study was approved by the Institutional Review Board of the Department of Psychology and the Center for Biomedical Imaging Research at Tsinghua University. All participants provided written informed consent before the experiments.

### Visual Stimuli

**FIGURE 1:**
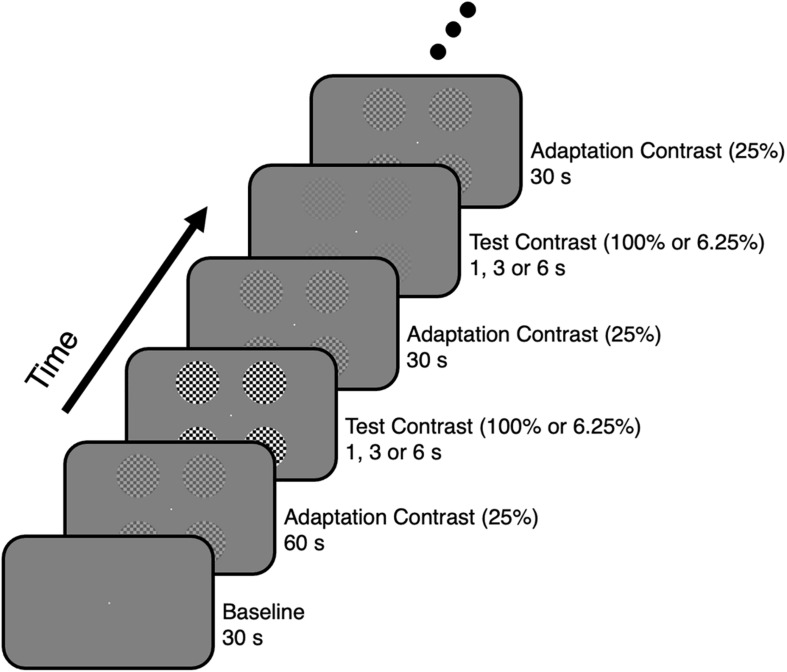
Visual stimulus presentation paradigm for the contrast adaptation experiment.

Four circular flickering checkerboards with different contrast levels were presented to elicit BOLD responses. The visual stimuli were displayed via a mirror and screen system. The visual stimuli were presented on an LCD screen, which was viewed through an angled mirror attached above the head coil. As there was an upgrade of the visual stimulus system during the research, an Invivo system (Gainesville, FL, United States) was used for the first four participants (screen size = 64 cm × 40 cm, corresponding visual angle ≈ 19.7° × 12.4°, resolution = 1280 × 800, refresh rate = 60 Hz) and a Sinorad system (Shenzhen, Guangdong, China) was used for other participants (screen size = 89 cm × 50 cm, corresponding visual angle ≈ 24.6° × 14.0°, resolution = 1920 × 1080, refresh rate = 60 Hz). The visual stimuli consisted of four circular flickering checkerboards (radius ≈ 2.0°, checker size ≈ 0.3° × 0.3°, flickering rate = 6 Hz), which were presented in four respective quadrants of a uniformly gray background with their centers ∼4.4° away from the center of the screen. Since it is difficult to map out the visual area boundaries corresponding to the fovea (Zeki, 1969; Dougherty et al., 2003; Schira et al., 2009; Wandell and Winawer, 2011) and the meridians that are sensitive to eye movements (Engel et al., 1997; Noory et al., 2015), the visual field corresponding to fovea as well as the horizontal and vertical meridians were avoided to constrain the BOLD responses inside V1. The checkerboards with a contrast level of 25% (Michelson contrast, adaptation contrast) were presented during the adaptation period. After this period, the checkerboards with a contrast level of 100 or 6.25% (Michelson contrast, test contrast) were presented to elicit respective BOLD signal increments or decrements (Figure 1). Increments and decrements of the contrast level were introduced to a logarithmic scale (in octaves, one step equals 2-fold), and a 4-fold contrast level change was used to elicit the largest BOLD signal increments or decrements (Gardner et al., 2005b). In addition to the four circular checkerboards, a circular fixation dot (radius ≈ 0.1°) was placed at the center of the screen throughout the whole experiment.

Contrast ramps were introduced when the stimuli changed their contrast level (Tuan et al., 2008). To be precise, checkerboards with intermediate contrast levels were presented sequentially during the change, and the contrast level would increase or decrease linearly as time went by. There were seven intermediate states for each change, and each intermediate state took 1/60 s.

### Experimental Design

All participants were instructed to complete a localizer experiment and a contrast adaptation experiment in the present study. The localizer experiment was conducted to identify the activated voxels. The localizer experiment consisted of two types of blocks which were a 30-s rest block and a 30-s stimulus block. The two blocks were presented and alternated four times in each run. No visual stimulus was presented on the gray background during the rest block, and checkerboards with a contrast level of 100% were presented during the stimulus block.

The event-related contrast adaptation experiment was performed to examine the linearity of BOLD responses (Figure 1). The contrast adaptation experiment consisted of three runs, with each run lasting for 890 s. For each run, no stimulus was presented at the background during the initial 30 s, then the adaptation stimulus with a contrast level of 25% was presented for 60 s. After the adaptation phase, 24 trials were conducted sequentially with each trial including a test stimulus presentation followed by a 30-s adaptation stimulus presentation. The test stimulus was presented for 1, 3, or 6 s, with a contrast level of 100 or 6.25%. Four repetitions for each combination of stimulus duration and contrast level were included in each run, and the order of trials was randomized.

Throughout the whole experiment, participants were instructed to conduct a button-pressing task. Every 1–5 s, the white fixation dot that the participants fixated on turned red for 200 ms, and participants were required to report the change of color with a button press within 1 s.

### MR Image Acquisition

Two different MRI systems were used during the experiment due to a mandatory upgrade of the system. A Philips Achieva 3 T MRI system was used for the first four participants and a Philips Ingenia CX 3 T MRI system was used for all other participants. Upon the MRI system upgrade, a serious artifact appeared in functional images using the new Philips Ingenia CX 3 T MRI system when the original Echo Planar Imaging (EPI) sequence that was used for the older Philips Achieva 3 T MRI system was applied to the new system. Therefore, a new EPI sequence with slightly different parameters was designed and applied under the guidance of professionals to adapt to the new Philips Ingenia CX 3 T MRI system. It should be noted that there were no systematic differences in the measurements between the new and old MRI systems.

The imaging data were recorded using a 32-channel radio-frequency coil. The whole-brain structural images were acquired using a T1-weighted Turbo Field Echo sequence (TR = 7.6 ms, TE = 3.7 ms, FOV = 23 × 23 cm^2^, in-plane voxel size = 0.96 × 0.96 mm^2^, in-plane matrix size = 240 × 240 pixels, 180 contiguous slices, slice thickness = 1 mm). Then the images of the slices that were perpendicular to the calcarine sulcus were collected, covering most areas of the occipital lobe extending from the occipital pole. The structural images for these slices were collected using a T1-weighted Turbo Field Echo sequence (TR = 2.2 s, TE = 13 ms, FOV = 23 × 23 cm^2^, in-plane voxel size = 0.45 × 0.45 mm^2^, in-plane matrix size = 512 × 512 pixels, 13–14 contiguous slices, slice thickness = 3 mm), and the functional images for these slices were acquired using an EPI sequence during the experiments (TR = 1 s, TE = 35 ms, flip angle = 90°, slice thickness = 3 mm). The EPI sequence that was used for the older Philips Achieva 3 T MRI system (FOV = 19.2 × 19.2 cm^2^, in-plane voxel size = 3 × 3 mm^2^, in-plane matrix size = 64 × 64 pixels, 13 contiguous slices, no Multi-band SENSE used) was slightly different from the EPI sequence that was used for the new Philips Ingenia CX 3 T MRI system (FOV = 22.0 × 22.0 cm^2^, in-plane voxel size = 2.75 × 2.75 mm^2^, in-plane matrix size = 80 × 80 pixels, 14 contiguous slices, Multi-band SENSE used, MB factor = 2). Lastly, additional empty scans were included at the beginning of each functional run, allowing the longitudinal magnetization to reach a steady state. The additional scans were excluded from the functional data that would be used for further analysis.

### Data Pre-processing

The functional images were head-motion corrected, followed by undergoing different pre-processing procedures for the localizer experiment and contrast adaptation experiment respectively. They were then registered to the structural images.

Firstly, head-motion correction was performed for the functional data via a two-step procedure: all EPI volumes were aligned to the first volume of the corresponding run and were then further aligned to the first volume of the time course of the localizer experiment. The *3dvolreg* program in AFNI^1^ (Cox, 1996) was used to conduct this procedure.

Secondly, the functional images for the localizer experiment were further pre-processed. Considering that the frequency range of BOLD signal fell between 0.0167 and 0.15 Hz (this frequency range was calculated based on the simulated BOLD time courses, see Supplementary Figure 1 for details), the linear trend for the BOLD time course was first removed, and a band-pass filter (high-pass frequency = 0.0125 Hz, low-pass frequency = 0.2 Hz) was then applied to remove the low and high-frequency noise. The *3dFourier* program in AFNI was used for temporal filtering, removing the mean and the linear trend before each filtering.

**FIGURE 2:**
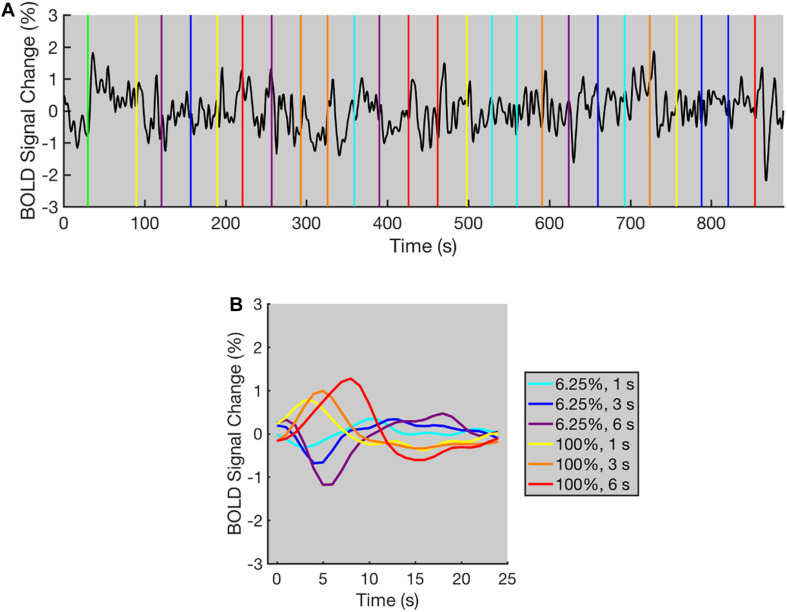
The typical BOLD time course for the contrast adaptation experiment (data from one representative selected voxel). **(A)** The BOLD time course for a single run. The green vertical line indicates the appearance of adaptation stimulus. All other colored vertical lines indicate the beginning of each trial, and the colors of vertical lines indicate the contrast level and duration of test stimuli. The time course displays positive and negative deflections following the contrast level increments and decrements in most trials. A low-pass filter (low-pass frequency = 0.2 Hz) was applied to the original time course for a clearer demonstration. **(B)** The averaged BOLD responses under each condition for all trials within three runs. The BOLD responses increased or decreased immediately after the contrast level increments or decrements, respectively. The colors of BOLD responses indicate the contrast level and duration of test stimuli.

Thirdly, the functional images for the contrast adaptation experiment were pre-processed by going through a series of procedures. As the initial period of the time course contained an ascending trend (the BOLD signal remained low in the first 30 s baseline, then increased to a higher level when the adaptation stimulus was presented, see Figure 2A), the linear trends of signal and noise were mixed together, and a complete removal of the linear trend would remove the signal that needed to be retained. Thus, the linear trends of the test period were removed after the first 90 volumes in each run were discarded. The simulation results suggested that the frequency range of the BOLD signal was approximately 0–0.15 Hz for the remaining time course in each run (Supplementary Figure 1), which was intermixed with the low-frequency noise. Instead of applying a band-pass filter on each run directly, a two-step filtering procedure was introduced. Specifically, the high-frequency noise was firstly removed with a low-pass filter (low-pass frequency = 0.2 Hz) in each run, followed by recombining the time courses of all trials of the same condition for three runs to form new time courses (Supplementary Figure 1). For instance, if a 1-s stimulus occurred 200 s after the start of a run, the trial for this stimulus that corresponded to volume 201–231 of this run would be selected to form the recombined time courses. The newly formed time courses with signals starting from the frequency that corresponded to the 12^th^ FFT component (12 cycles per time course) were then filtered with a high-pass filter, in which the high-pass frequency that equaled the frequency of the 10^th^ FFT component was applied to the newly formed time courses to remove the low-frequency noise. The frequency of the 10^th^ FFT component was calculated according to the time length of the newly formed time course. Each recombined time course contained the time courses of 12 trials, and a total of 6 recombined time courses were formed for the 6 conditions (2 BOLD response type × 3 stimulus duration), respectively. As each trial contained a test stimulus presentation followed by a 30-s adaptation stimulus presentation, the time length for the newly formed time courses under the 1-s stimulus condition was 31 × 12 = 372 s, and the frequency of the 10^th^ FFT component was 10 cycle / 372 s = 0.0269 Hz, which would be applied to remove the low-frequency noise. After the temporal filtering procedure, the measured BOLD responses of the six conditions were calculated for every voxel by averaging the time courses of the 12 trials. To unify the time length of the measured responses across all conditions, the first 25 volumes (corresponding to 25 s) of the measured responses were used in the linearity analysis, signal-to-noise ratio (SNR) analysis, and for calculating *r*^2^ of the contrast adaptation experiment (see “Linearity Analysis,” “Signal-to-noise Ratio (SNR) Analysis,” and “Activated Voxels Selection” sections).

Finally, the functional images were registered to the high-resolution structural images using the mrAlign tool in mrTools (Gardner et al., 2018). The first volume for the functional images of the localizer experiment was registered to the structural images for the corresponding slices, where these structural images were further registered to the whole brain structural images.

In addition, all other pre-processing procedures were conducted by custom programs written in MATLAB R2016b (Mathworks, Natick, MA, United States). The programs (i.e., cbiReadNifti.m and cbiWriteNifti.m) in mrTools (Gardner et al., 2018) were used to read and write NIfTI files.

### Activated Voxels Selection

To choose voxels that were significantly activated by the visual stimuli within V1, the activated voxels were selected following three steps for each participant (Gardner et al., 2005b): (1) the boundaries of V1 were drawn based on a separate retinotopy experiment; (2) the voxels that were significantly activated by the visual stimuli were determined using the BOLD time courses of a block design localizer experiment (see “Experimental Design” section for the procedure of localizer experiment); (3) combining the results of the previous two steps, clusters of significantly activated voxels were chosen within V1. The result of the contrast adaptation experiment was not involved in the selection of activated voxels, avoiding the interaction between activated voxel selection and linearity analysis.

The boundaries of V1 in each participant were determined in a separate retinotopy experiment using the traveling-wave method (Engel et al., 1994, 1997; Wandell et al., 2007). Six runs of rotating wedge stimuli were presented to map retinotopic organization with respect to visual polar angle, and four runs of moving ring stimuli were presented to map retinotopic organization with respect to visual eccentricity. Each run took 252 s and contained 10.5 cycles. After removing the imaging data for the first 0.5 cycles of each run, the BOLD time courses for the runs with the wedge stimuli and ring stimuli presented were averaged separately. Subsequently, the averaged time courses in each voxel were fitted to the sinusoids at the stimulus frequency. A high correlation between the actual and fitted time courses suggested that the voxel was more likely to be within the visual cortex. In addition, the fitted sinusoids for some voxels with high correlation reached their peak value when the visual stimulus appeared at vertical meridians, and the boundaries of V1 were drawn on these voxels on the flattened cortical surfaces. The data analysis was conducted with mrTools, and the detailed data analysis procedures are available online^2^. The flattened cortical surfaces were created by mrTools based on the segmentation done by FreeSurfer^3^ (Fischl, 2012).

To determine significantly activated voxels, an index (*r*^2^) was calculated for each voxel’s time course that was acquired from the localizer experiment, indicating what percentage of the variance in the time course was accounted for by the visual stimuli (Gardner et al., 2005b). To calculate *r*^2^, an HRF was first estimated for each voxel’s time course using a deconvolution method (also called the finite impulse response model) (Dale, 1999; Hinrichs et al., 2000). Based on our previous study (Gardner et al., 2005b), the deconvolution method was chosen instead of the generally used GLM method as it does not presume any particular shape for the time course of the HRF. The goodness-of-fit (*r*^2^ in this study) was then estimated, which is equal to the percentage of variance in the original time course that is accounted for by the estimated time course. The estimated time course was the convolution of the estimated HRF and the time course of the visual stimuli. The value of *r*^2^ ranges from 0 to 1. The higher the value of *r*^2^, the more variance was accounted for by the estimated time course. The value of *r*^2^ is equal to 0 if the estimated time course does not account for any of the variance, and the value of *r*^2^ is equal to 1 if the estimated time course accounts for all the variance. The calculation of *r*^2^ was implemented by mrTools^4^. To determine the significance level of a certain *r*^2^ value, the *r*^2^ distributions of randomized time courses were constructed. For each participant, the BOLD time courses for all voxels in retinotopically defined V1 were extracted after the head-motion-correction procedure. This was followed by random shuffling of the time points in each BOLD time course, creating 100000 randomized time courses in total. After these randomized time courses went through the pre-processing procedures (i.e., removing the linear trend, filtering), the values of *r*^2^ were computed for each time course, and these values were used to form the randomized distribution of *r*^2^. The false positive rate for the value of *r*^2^ for each voxel was determined based on the randomized distribution. For example, if the value of *r*^2^ was ranked as the 99.9% highest in the randomized distribution, then its false positive rate would be equal to 0.001. The voxels that had a false positive rate below 0.001 were shown for one of the participants in Supplementary Figure 2. The false positive rates for the voxels in V1 were corrected using the false discovery rate (FDR) approach (Benjamini and Hochberg, 1995). A voxel was considered significantly activated when its corrected false positive rate was smaller than 0.001. Another randomized distribution using simulated time courses with temporal autocorrelation was generated to verify the reliability of this result (see Supplementary Material). When computing the randomized distributions, programs (i.e., cbiReadNifti.m and cbiWriteNifti.m) in mrTools were applied to read and write NIfTI files, while all other processes were conducted by custom programs written in MATLAB R2016b.

Clusters of significantly activated voxels were selected within V1 of each participant. The size of clusters was defined based on the size of the visual stimuli according to previous studies (Horton and Hoyt, 1991; Sereno et al., 1995). In addition, the visual stimuli in the localizer experiment elicited a BOLD signal increment in all selected voxels. Furthermore, the *r*^2^ values for every selected voxel were calculated using the time courses of the contrast adaptation experiment to verify that the selected voxels were responding to the visual stimuli in the contrast adaptation experiment. The result showed that 98.7% of the selected voxels had their *r*^2^ value above the chance level (false positive rate = 0.001), suggesting that most selected voxels responded to the visual stimuli in the contrast adaptation experiment.

### Linearity Analysis

To obtain a voxel-wise test of linearity, the similarity between measured BOLD responses and their linear predictions was assessed in every selected voxel by calculating the value of a similarity index, the Dice index (Dice, 1945; Cha, 2007). The linear predictions for the measured BOLD responses were calculated by adding the BOLD responses to the shorter stimuli with their temporally shifted copies (Boynton et al., 1996). For instance, to predict the BOLD signal increment to the 6-s stimulus, the BOLD signal increment to the 3-s stimulus was summed up with the copy of its shifted self by 3 s. In the present study, the linear predictions of responses to the 3-s stimulus were made by responses to the 1-s stimulus, and the linear predictions of responses to the 6-s stimulus were made by responses to the 1-s stimulus and 3-s stimulus, respectively. It is important to underline that the linear prediction should approximate measured BOLD response when the linearity holds, whereas deviations should occur when the BOLD responses behave in a non-linear manner. Therefore, the more the BOLD response behaved in a linear way, the higher the degree of similarity between the measured BOLD response and its linear prediction. Previous studies suggested that the difference in the amplitude of BOLD time courses was the major indicator of non-linearity (Birn et al., 2001; Soltysik et al., 2004). Hence, the Dice index was chosen as the suitable indicator as it is sensitive to the difference in the amplitude between time courses. Other similarity indices (e.g., the correlation coefficient) tend to describe the similarity in the direction of vectors, thus they can only describe the similarity in the shape but not the amplitude between time courses, making them unsuitable for the present study. Assuming a measured BOLD response (M) and its linear prediction (P) with n time points, their Dice index was calculated using the formula below (the subscript t refers to the t^th^ time point):

(1) $${\text{s}}_{\text{Dice}}=\frac{2\sum_{\text{t}=1}^{\text{n}}{\text{P}}_{\text{t}}{\text{M}}_{\text{t}}}{\sum_{\text{t}=1}^{\text{n}}{\text{P}}_{\text{t}}^{2}+\sum_{\text{t}=1}^{\text{n}}{\text{M}}_{\text{t}}^{2}}$$

The value of the Dice index ranges from −1 to 1: the value is equal to 1 if and only if P = M, and the value is equal to −1 if and only if P = −M. The value increases if the similarity between P and M increases. Wilcoxon signed ranks tests were performed to assess whether there was a difference in the value of the Dice index across the BOLD response types and stimulus durations. In these tests, one data point referred to the Dice index of one selected voxel. These tests were implemented by the “signrank” function in MATLAB R2016b. To assess how many voxels behaved in a linear manner, the percentages of voxels that had their Dice index above chance level (false positive rate = 0.05) were calculated under each condition. The Dice thresholds for chance level were calculated based on the randomized distributions that were established by computing the Dice index for randomly shuffled time courses (see “Activated Voxels Selection” section for randomly shuffled time courses).

The voxel-wise overestimation and underestimation patterns were examined by estimating the contrast index for the amplitude of the HRFs in each selected voxel (Birn et al., 2001; Soltysik et al., 2004). HRFs were fitted to the measured BOLD responses under each condition, then the contrast index for the amplitude of the HRFs was calculated across stimulus durations of the BOLD signal increments and decrements, respectively. Firstly, a two-gamma HRF was fitted to the measured BOLD responses under each of the six conditions (see “Data Pre-processing” section for measured BOLD responses). The measured BOLD responses were either increment (for the 100% contrast condition) or decrement (for the 6.25% contrast condition), thus the HRF was either a positive HRF or a negative HRF. The positive HRF was modeled by a canonical HRF, and the negative HRF was modeled by zero minus canonical HRF. The formula for canonical HRF is given below (Lindquist et al., 2009):

(2) $$\begin{array}{c} \text{h}\left(\text{t}\right)=\\ \left\{ \begin{array}{cc} \text{A}\left(\frac{{\left(\text{t}-{\text{t}}_{\text{onset}}\right)}^{\alpha_{1}-1}\beta_{1}^{\alpha_{1}}{\text{e}}^{-\beta_{1}\left(\text{t}-{\text{t}}_{\text{onset}}\right)}}{\Gamma \left(\alpha_{1}\right)}-\text{c}\frac{{\left(\text{t}-{\text{t}}_{\text{onset}}\right)}^{\alpha_{2}-1}\beta_{2}^{\alpha_{2}}{\text{e}}^{-\beta_{2}\left(\text{t}-{\text{t}}_{\text{onset}}\right)}}{\Gamma \left(\alpha_{2}\right)}\right), & \\ \text{t}\geq {\text{t}}_{\text{onset}} & \\ 0,\text{t}<{\text{t}}_{\text{onset}} & \end{array}\right. \end{array}$$

The two α parameters were replaced by μβ + 1 in practice, where μ is the time each Gamma function reaches its peak. The formula has seven free parameters with ranges restricted to obtain a reasonable fit: μ_1_ (from 0.5 to 8), μ_2_ (from 4 to 16), β_1_ (from 0 to 5), β_2_ (from 0 to 5), c (from 0 to 1), t_onset_ (from 0 to 5), and A (from 0 to 20). After convolving the HRF with the time course of the visual stimuli (which was modeled as a boxcar function), the time course was fitted to the measured response using the trust-region-reflective algorithm (via “lsqcurvefit” function in MATLAB R2016b). An HRF with more flexible parameters was also applied to prove that the deviation patterns did not result from the restricted parameters. The ranges of parameters for the flexible HRF are μ_1_ (from 0 to 24), μ_2_ (from 0 to 24), β_1_ (from 0 to positive infinity), β_2_ (from 0 to positive infinity), c (from 0 to 1), t_onset_ (from 0 to 24), and A (from 0 to positive infinity). Secondly, the amplitude of HRF was estimated as the maximum of positive HRF or the minimum of negative HRF (note that both the maximum and minimum represent the peak of HRFs, thus the undershoot or overshoot after the peak was not considered). Thirdly, the contrast index for the amplitude of HRFs across stimulus durations was calculated for the BOLD signal increments and decrements separately. The formula for the contrast index is given below:

(3) $$\text{Contrast}=\frac{{\text{Amp}}_{\text{short}}-{\text{Amp}}_{\text{long}}}{{\text{Amp}}_{\text{short}}+{\text{Amp}}_{\text{long}}}$$

The amplitude of the HRFs for the short stimulus (Amp_short_) was compared with the amplitude of HRFs for the longer stimulus (Amp_long_). To be precise, the amplitude of the HRFs for the 3-s stimulus condition was compared with the amplitude of the HRFs for the 6-s stimulus condition, and the amplitude of the HRFs for the 1-s stimulus condition was compared with the amplitude of the HRFs for the 3-s and 6-s condition. The HRF for the short stimulus corresponded to the linear prediction, whereas HRF for the longer stimulus corresponded to the measured BOLD responses. Note that in the event when the BOLD response behaved in a linear manner, the linear prediction should approximate the measured BOLD responses, which yielded similar HRFs, and the contrast index should be close to 0. On the contrary, when an overestimation (or underestimation) pattern occurred, the contrast index should be above (or below) 0. The percentage of voxels that had a contrast index above 0 was calculated under each condition. For each distribution of the contrast index, a Kolmogorov–Smirnov one-sample test was performed to assess if the distribution significantly deviated from a normal distribution with its mean equaled to 0 and with the same standard deviation as the distribution for the contrast index. This test was implemented by the “kstest” function in MATLAB R2016b, and one data point in this test referred to the contrast index of one selected voxel. Like previous studies that used the ratio for the amplitudes of the BOLD responses to assess the linearity (Birn et al., 2001; Birn and Bandettini, 2005), the HRF amplitude ratios across stimulus durations were also calculated for the BOLD signal increments and decrements separately. To calculate the HRF amplitude ratio, the amplitude of the HRFs for the short stimulus was divided by the amplitude of HRFs for the longer stimulus. It should be noted that the distribution of the HRF amplitude ratio is asymmetric and unbounded for positive values, while the distribution of the contrast index is symmetric and ranges from −1 to 1, making the contrast index more appropriate for the statistical analysis. Therefore, the contrast index was used as the main indicator of the deviation pattern in the present study.

### Signal-to-Noise Ratio (SNR) Analysis

To support the validity of linearity analysis, the deviation pattern across the high SNR group and low SNR group were compared for the BOLD signal increments and decrements separately. For each selected voxel, the pre-processed time courses of all trials for the BOLD signal increments and decrements of three runs were recombined separately, and the SNR was calculated for each of the recombined time courses. The time course of each trial was unified to the first 25 volumes (seconds) starting from stimulus presentation, which yielded a task frequency of 25 s per cycle. The SNR was calculated by dividing the magnitude of the BOLD signal at task frequency (0.04 Hz) to the mean magnitude of the BOLD signal at 0.33–0.50 Hz, which represented the frequency range for high-frequency noise (Sun et al., 2013; Kuriki et al., 2015). A Wilcoxon signed ranks test was performed to compare SNR across BOLD signal increments and decrements. To explore the effect of SNR, the selected voxels were divided into a low and a high SNR group by the median SNR (two different divisions were created for the BOLD signal increments and decrements separately). Kolmogorov–Smirnov tests were performed to assess if the distributions of the contrast index differed across high SNR and low SNR groups using the “kstest2” function in MATLAB R2016b. In these tests, one data point referred to the contrast index of one selected voxel. Other noise bands (e.g., all frequencies except the task frequency) were also applied to prove that the choice of noise bands did not affect the deviation patterns in two SNR groups.

## Results

A total of 223 voxels were selected from 12 healthy participants. The number of voxels selected from each participant varies from 12 to 26. The response accuracy of the button-pressing task was 99.4% ± 1.0% and 98.4% ± 1.7% (mean accuracy ± standard deviation across participants) for the localizer experiment and contrast adaptation experiment, respectively, confirming that the participants maintained fixation throughout the whole experiment.

The BOLD time course of one representative selected voxel is shown in Figure 2. The BOLD time course was relatively stable during the initial 30 s of each run. Then, a rapid increase occurred immediately after the adaptation stimulus was presented (Figure 2A). During the 60-s adaptation phase, the time course slowly decayed to a lower level, showing a similar trend to the typical time course for adaptation as observed in previous studies (Gardner et al., 2005b). After the adaptation phase, the time course displayed transient peaks and dips corresponding to the increments and decrements of contrast level respectively in most trials. To combine the fMRI data from three runs, the mean BOLD signal level for each run was calculated after removing the first 90 volumes (duration = 90 s, see “Data Pre-processing” section for details), and the percent signal changes were calculated relative to the mean BOLD signal level. The measured BOLD responses were then computed by averaging the time course of percent signal changes for all trials under the same condition of three runs. As shown in Figure 2B, immediately after the contrast level increments or decrements, the measured BOLD responses in a representative selected voxel increased or decreased, respectively, indicating that the BOLD signal increments and decrements were successfully elicited using the contrast adaptation paradigm.

**FIGURE 3:**
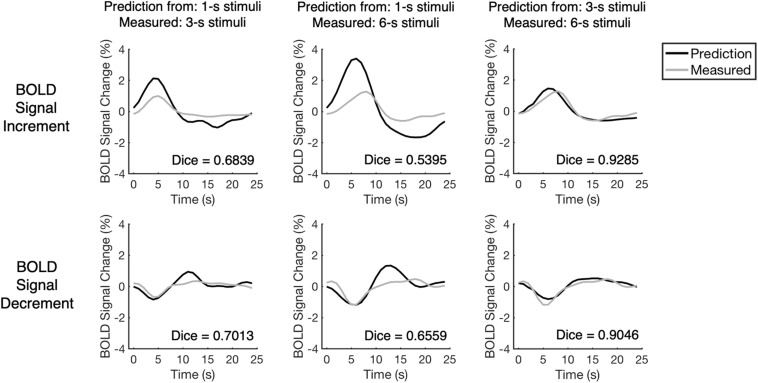
The comparison between measured BOLD responses and their linear predictions for one representative voxel. The voxel is the same as the one presented in Figure 2. The BOLD responses and their temporally shifted copies were added up linearly to predict the BOLD responses to longer stimuli. As the measured BOLD responses and their linear prediction have high similarity, the performances of BOLD responses to longer stimuli were close to linear. The Dice index is a similarity index that ranges from –1 to 1 in which, higher Dice index indicates higher similarity between measured BOLD response and its linear prediction.

We examined the linearity of BOLD responses via assessing the similarity between measured BOLD responses and their linear predictions (see “Linearity Analysis” section for detailed methods). Take the representative voxel for example, the performances of BOLD responses to longer stimuli were closer to linear: the linear predictions made by BOLD responses to 3-s stimuli approximated the BOLD responses to 6-s stimuli, whereas the linear predictions made by BOLD responses to 1-s stimuli deviated more from the BOLD responses to longer stimuli (Figure 3). To demonstrate a more typical pattern, the mean measured BOLD responses for all selected voxels from all participants were also calculated and compared with their linear predictions (Supplementary Figure 3).

**FIGURE 4:**
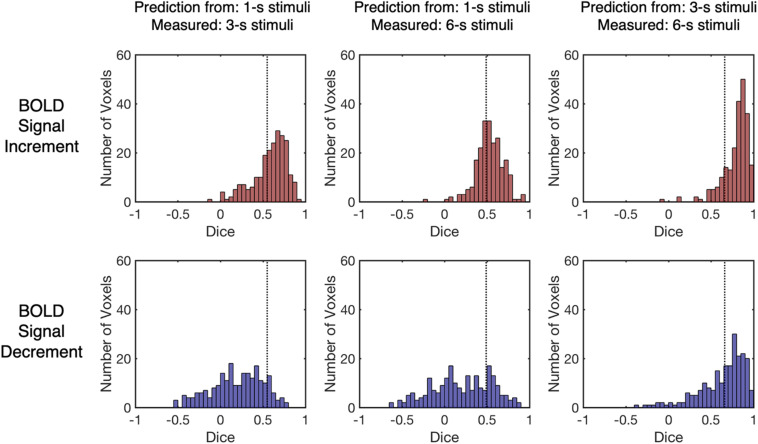
The distributions of the Dice index for different BOLD response types and stimulus durations. The Dice index describes the similarity between measured BOLD response and its linear prediction. The vertical dashed lines indicate the upper thresholds for chance levels (false positive rate = 0.05, one-tailed, corrected), and having a Dice index above chance level suggests that the response to the short stimulus can predict response to the longer stimulus in a linear way. Each data point refers to the Dice index of one selected voxel. Each distribution includes all selected voxels from all participants (same for the figures below).

To evaluate the similarity quantitatively, the Dice index was calculated (Dice, 1945; Cha, 2007) for each pair of the measured BOLD response and its linear prediction. The Dice index describes the similarity between two vectors, in which the value of the Dice index increases when the similarity increases (thus more similar time courses tend to have a higher Dice index, see Figure 3). The distribution of the Dice index of all selected voxels from all participants (*n* = 223) varied across the BOLD response types and stimulus durations (Figure 4). The value of the Dice index for the BOLD signal increments was higher than that for the BOLD signal decrements under all stimulus durations (Wilcoxon signed ranks test, *Z* > 7.4, *p* < 0.001, two-tailed, corrected, Supplementary Table 1). For both BOLD signal increments and decrements, the responses to the 3-s stimuli predicted measured responses in a more linear way in comparison to responses to the 1-s stimuli, showing a higher value of Dice index (Wilcoxon signed ranks test, *Z* > 11.5, *p* < 0.001, two-tailed, corrected, Supplementary Table 2) and having more voxels with the Dice index above chance level (Figure 4 and Supplementary Table 3; the thresholds for chance levels were defined using the Dice index distributions for randomly shuffled time courses, corresponding to false positive rate = 0.05, one-tailed, corrected).

**FIGURE 5:**
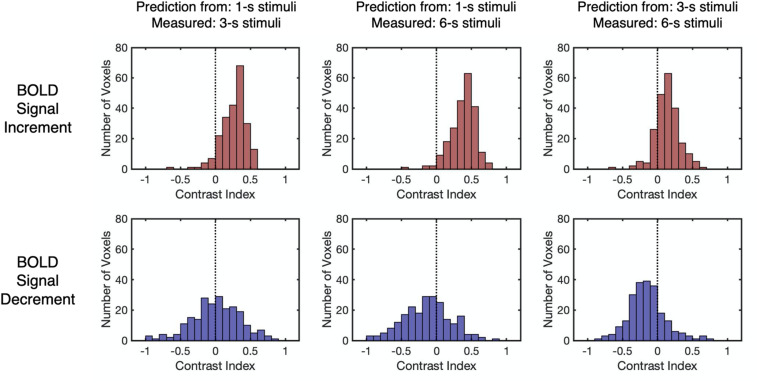
The distributions of the contrast index for different BOLD response types and stimulus durations. The contrast index is close to 0 when linear prediction and measured response have similar amplitudes and is larger (or smaller) than 0 when linear prediction overestimates (or underestimates) measured response. The vertical dashed lines indicate that the contrast index equals 0. Each data point refers to the contrast index of one selected voxel.

As the Dice index merely describes the similarity between the predicted and the measured BOLD responses without knowing the deviation patterns (i.e., underestimation or overestimation), the contrast index for the amplitude of HRFs was computed across all stimulus durations to depict the deviation patterns under each condition in all selected voxels (see “Linearity Analysis” section for detailed methods). The contrast index should be close to 0 if the linear prediction and measured response have similar amplitudes, larger than 0 if the linear prediction overestimates the measured response, and smaller than 0 if underestimation exists. As shown in Figure 5, the distributions of the contrast index for all selected voxels from participants (*n* = 223) varied across conditions. The distributions of the contrast index were compared with normal distributions with their mean values equaling 0 and the same standard deviation as the distributions of the contrast index in every condition. Except for the contrast index for the BOLD signal decrements to 1-s stimulus and decrements to 3-s stimulus (Kolmogorov–Smirnov one-sample test, *D* = 0.048, *p* = 0.999, unequal, corrected), the distributions of the contrast index were significantly different from the normal distributions in every condition (Kolmogorov–Smirnov one-sample test, *D* > 0.173, *p* < 0.001, unequal, corrected, Supplementary Table 4). The BOLD responses to the 3-s stimuli predicted measured responses in a nearly linear manner, showing slight overestimation and underestimation tendencies corresponding to the BOLD signal increments and decrements, respectively (Figure 5 and Supplementary Table 5). While the signal increments to a 1-s stimulus demonstrated a consistent overestimation pattern, the pattern of signal decrements ranged from underestimation to overestimation (Figure 5 and Supplementary Table 5). The distributions of the HRF amplitude ratio also demonstrated similar deviation patterns (Supplementary Figure 4). In addition, the distributions of the contrast index for the selected voxels in each participant were computed, showing similar deviation patterns that were observed in all selected voxels (Supplementary Figures 5, 6). To verify that the deviation patterns did not result from the restricted parameters in the HRF, time courses were fitted with a more flexible HRF. The resulting deviation patterns were found to be similar to the distributions presented in Figure 5 (Supplementary Figure 7), suggesting that the range of the parameters for the HRF did not affect the deviation patterns. Moreover, as the adaptation stimulus might affect the baseline of the BOLD responses to the test stimuli, the linearity analysis was conducted after subtracting the fMRI signal of the last volume before each trial in the pre-processing stage to ensure there was no influence from the adaptation stimulus on the results of linearity analysis. The results of this analysis were similar to the result of the original analysis (Supplementary Figure 8), which supported the idea that the results of the linearity analysis were not influenced by the effect of the adaptation stimulus. Furthermore, the eccentricity and SNR were plotted versus the amplitude of HRF to test if there was systematic large-scale bias in the responses (see Supplementary Figure 9 for details).

**FIGURE 6:**
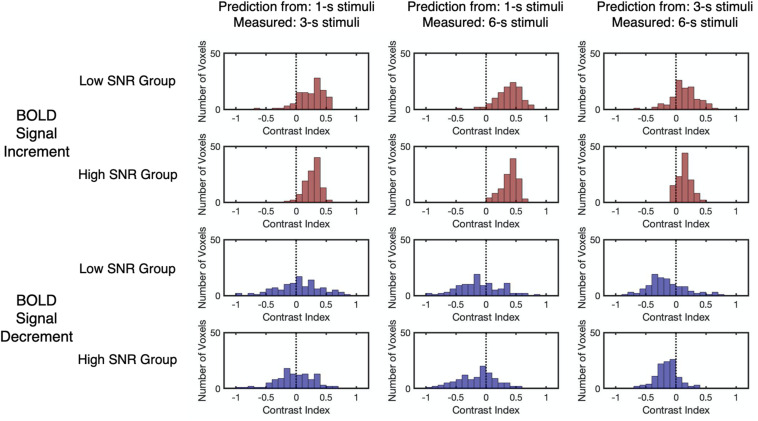
The distributions of the contrast index for the low SNR group and high SNR group. The low SNR group (*n* = 111) and high SNR group (*n* = 112) were split by the median *r*^2^ of the corresponding BOLD response type. The vertical dashed lines indicate that the contrast index equals 0. Each data point refers to the contrast index of one selected voxel.

The relationship between the contrast index and SNR was examined to confirm the validity of the linearity analysis. Results showed that the SNR for BOLD signal decrements was significantly lower than those for the BOLD signal increments (*Z* = 11.98, *p* < 0.001, two-tailed, Supplementary Figure 10). To evaluate the effect of SNR on the contrast index, all selected voxels from participants were divided into either a low SNR group (*n* = 111) or a high SNR group (*n* = 112), with reference to the median SNR of the corresponding BOLD response type (Figure 6 and Supplementary Table 6). The results reflected no significant difference in the distributions of the contrast index across the SNR groups under all conditions (Kolmogorov–Smirnov two-sample test, *D* < 0.19, *p* > 0.05, unequal, corrected, Supplementary Table 7). While the BOLD signal increments showed a consistent overestimation pattern, the linear predictions made by signal decrements to the 1-s stimulus showed an underestimation-to-overestimation pattern, and the predictions made by the signal decrements to the 3-s stimulus showed a consistent underestimation pattern. To confirm that the choice of noise bands did not affect the deviation patterns in two SNR groups, other noise bands (e.g., all frequencies except the task frequency) were also applied for further examination. The resulting observation showed that using other noise bands did not affect the deviation patterns in two SNR groups (Supplementary Figure 11). As the SNR for the BOLD signal decrements were significantly lower than those for the BOLD signal increments, the underestimation-to-overestimation pattern might be a result of the low SNR. Therefore, the deviation patterns for the low SNR group for the BOLD signal increment and the high SNR group for the BOLD signal decrement were further examined as the two groups had similar distributions of SNR (Kolmogorov–Smirnov two-sample test, *D* = 0.13, *p* = 0.25). The observed deviation patterns for the time courses of the two groups were the same as the pattern for all selected voxels (Figure 6), showing that the low SNR for the BOLD signal decrements might not be the main reason for the underestimation-to-overestimation pattern.

## Discussion

The present study examined the linearity for both BOLD signal increments and decrements at the voxel-wise level using a contrast adaptation paradigm. Results suggested that linearity was violated for the BOLD signal increments and decrements to the transient visual stimuli, and different deviation patterns were found across BOLD response types, stimulus durations, and voxels. The deviation patterns for both low and high SNR groups were consistent with the general patterns for all selected voxels, suggesting that SNR was not the main cause of the present deviation pattern.

In the present study, a contrast adaptation paradigm (Gardner et al., 2005b) was conducted to compare the BOLD signal increments and decrements under the same experimental settings. Previous studies on the linearity of BOLD signal decrements have chosen different baseline conditions for the BOLD signal increments and decrements (Birn and Bandettini, 2005; Tang et al., 2009). In these studies, the BOLD signal increments were elicited by the presentation of visual stimulus with the baseline condition being a blank screen condition; the BOLD signal decrements were elicited by the cessation of visual stimulus with the baseline condition being a the visual stimulus presenting condition. Therefore, factors related to the different baseline conditions might influence the result of linearity analysis (Ohzawa et al., 1982; Stark and Squire, 2001). With reference to our previous study (Gardner et al., 2005b), the contrast adaptation paradigm was used in the present study to unify the baseline condition for both BOLD signal increments and decrements, providing the possibility to make a direct comparison between them. This baseline condition that presents the visual stimulus for adaptation has been employed in past contrast adaptation studies (Kohn, 2007), with results proving that the BOLD signal increments and decrements could be induced effectively using the contrast adaptation paradigm (Gardner et al., 2005b).

The results of the linearity analysis for the BOLD signal increments are consistent with previous studies. Concerning prior studies for BOLD signal increments, the BOLD signal increments to a relatively longer stimulus behaved in a linear way, whereas a robust overestimation pattern was found when the BOLD signal increments had stimulus durations of less than 3-s in the human visual cortex (Boynton et al., 1996; Friston et al., 1998; Vazquez and Noll, 1998; Birn et al., 2001; Soltysik et al., 2004; Wager et al., 2005). The present study found that when undergoing a longer stimulus, the linear predictions made by BOLD signal increments to the 3-s stimulus approximated to the BOLD signal increments, whereas the linear predictions made by the BOLD signal increments to the 1-s stimulus deviated more from the BOLD signal increments, showing a consistent overestimation pattern in almost all voxels.

Previous studies explained the physiological origin of the overestimation pattern for the BOLD signal increment from the perspective of neural activity and hemodynamic activity. It is generally believed that the BOLD signal increment was elicited via two steps: the stimulus first elicits an increase in the localized neural activity, then the increased neural activity induced an oversupply of oxygenated blood (with changes in the cerebral blood flow, cerebral blood volume, and cerebral metabolic rate for oxygen), which decreased the localized ratio of deoxyhemoglobin to oxyhemoglobin through a complicated hemodynamic transformation (Ogawa et al., 1992; Deyoe et al., 1994; Heeger and Ress, 2002). Therefore, the overestimation pattern could arise from the stimulus-induced neural activity, the hemodynamic transformation, or both. Comparing with longer duration stimuli, the neural activity at the onset and offset of the transient stimuli contributes more to the averaged neuronal activity. Thus, in the case of the transient stimuli, the relatively high neural activity at the onset and offset of the stimuli can lead to stronger averaged neuronal activity, causing an overestimation pattern in the linearity analysis (Boynton et al., 1996; Birn et al., 2001; Soltysik et al., 2004). Other studies also stated that the overestimation pattern could result from the hemodynamic transformation from neural activity to BOLD responses. Previous studies suggested that the non-linearity of BOLD signal increments could be modeled by modifying components that were related to hemodynamic changes in the Buxton’s balloon model (Buxton et al., 1998; Glover, 1999; Friston et al., 2000; Obata et al., 2004). Experimental results indicated that there was a non-linear relationship between the underlying hemodynamic changes (e.g., cerebral blood flow, cerebral metabolic rate of oxygen) and the BOLD responses (Rees et al., 1997; Moradi and Buxton, 2013). The macro-vascular activity was also deemed as a potential cause of non-linearity, as the removal of voxels from the large vessels or the suppression of large-vessel contribution could strongly decrease the non-linearity of the BOLD signal increments (Zhang et al., 2008, 2009). The explanations from both perspectives were supported by experimental evidence, and it was argued that they both contributed to the non-linearity of BOLD signal increments (Miller et al., 2001; Buxton et al., 2004; Tuan et al., 2008).

Previous studies found an underestimation pattern in the BOLD signal decrements and explained this pattern from the perspective of neural activity and hemodynamics (Birn and Bandettini, 2005; Tang et al., 2009). Birn and Bandettini (2005) simulated the BOLD signal decrements by applying a combination of neural adaptation, neural refractory effects, and neural responses to stimulus offsets. The simulated BOLD signal decrements demonstrated an underestimation pattern that was similar to the measured BOLD signal decrements. Tang et al. (2009) modified the balloon model, assuming that there were different blood flow-in time constants for stimulus onsets and offsets. The measured BOLD signal decrements could be well modeled by applying this change, and the results of linearity analysis for both BOLD signal increments and decrements could be predicted.

Compared with these earlier findings, an underestimation-to-overestimation pattern was revealed for the BOLD signal decrements to the 1-s stimulus for the first time. Former studies usually averaged the BOLD signal decrements from all selected voxels in the primary visual cortex (Birn and Bandettini, 2005) or across participants (Tang et al., 2009). In contrast, the present study focused on the voxel-wise results and found various deviation patterns for BOLD signal decrements. Specifically, the BOLD signal decrements to the 3-s stimulus demonstrated an underestimation pattern, while the deviation pattern for the BOLD signal decrements to the 1-s stimulus varied from underestimation to overestimation. More importantly, this voxel-wise variation in the deviation pattern could not be observed by the linearity analysis for averaged BOLD responses, showing the benefit of voxel-wise analysis. Furthermore, the deviation patterns of both BOLD signal increments and decrements did not differ across low and high SNR groups, suggesting that the SNR was not the main cause of the deviation pattern.

The underestimation-to-overestimation pattern found in the signal decrements mentioned above may be attributed to the underlying physiological processes. Previous results suggested that the BOLD signal decrements that were elicited in the contrast adaptation paradigm may have a neural origin (Ohzawa et al., 1982; Gardner et al., 2005b), making it reasonable for researchers to interpret the present results from the neural activity perspective. Past studies revealed neural activity bursts at the onset and offset of visual stimulus (Albrecht et al., 1984; Maddess et al., 1988; Duysens et al., 1996), which could increase the amplitude of BOLD signal increments and decrease the amplitude of BOLD signal decrements (Birn and Bandettini, 2005; Tuan et al., 2008), leading to the overestimation pattern for BOLD signal increments and underestimation pattern for BOLD signal decrements. Likewise, neuronal habituation might also contribute to the deviation patterns of the BOLD responses. More specifically, the neuronal habituation suggests that neural activity will get closer to the baseline level as the stimulus persists (Ohzawa et al., 1985; Logothetis et al., 2001; Bandettini, 2014), thus the BOLD signal decrements to a shorter stimulus possess larger amplitudes, and an overestimation pattern is predicted. These two effects may combine and give rise to the deviation patterns in linearity for the BOLD signal decrements. Additionally, the physiological mechanism of BOLD signal decrements remains unclear (Birn and Bandettini, 2005; Tang et al., 2009), opening these complicated hemodynamic mechanisms to all possible explanations.

BOLD fMRI measures neural activity through complicated hemodynamic changes: the BOLD response is determined by the localized changes in blood flow, blood volume, and metabolic rate of oxygen, reflecting the impact of localized neural activity (Ogawa et al., 1992; Deyoe et al., 1994; Heeger and Ress, 2002). The linearity of BOLD responses suggests a direct relationship between the neural activity and BOLD responses, making the BOLD signal a good indicator of neural activity. Numerous studies applied BOLD fMRI to infer neural activity, with corresponding data analysis procedures already presumed in regard to the linearity of BOLD responses (Friston et al., 1994a, b; Boynton et al., 2012; Poline and Brett, 2012). Therefore, testing the linearity of BOLD responses is necessary for both understanding the physiological nature of BOLD responses and proving the robustness of analysis procedures. Even if the linearity test yields a negative result, it could promote the development of analysis that is suitable for new circumstances. For instance, to account for the overestimation pattern found in BOLD signal increment to transient stimuli, new analysis procedures were developed based on the experimental results by applying filtering functions (Vazquez and Noll, 1998), Volterra series (Friston et al., 1998), and other modeling tools (Wager et al., 2005). The present study tested the linearity of BOLD signal decrements in a voxel-wise manner, shedding new light on the analysis of the BOLD signal decrement.

### Limitations

Limited by the length of the experiment, only three contrast levels (100, 25, and 6.25%) were chosen for the present study. The three contrast levels were chosen based on a logarithmic scale (Ohzawa et al., 1985), maximizing the difference between the contrast levels. Future studies are advised to apply more contrast levels in the experiment which can help to better understand the relationship between the stimulus intensity and the linearity of BOLD responses.

While previous studies used the averaged time courses for linearity analysis (Birn and Bandettini, 2005; Tang et al., 2009), the present study used the time course of each selected voxel. Although the BOLD signal increment showed a consistent overestimation pattern, the BOLD signal decrement showed an underestimation-to-overestimation pattern across the time courses of the selected voxels, which was different from the underestimation that was revealed in the averaged time courses. As the SNR of BOLD signal decrement was lower than the SNR of BOLD signal increment, the underestimation-to-overestimation pattern in the BOLD signal decrement could potentially be a result of the low SNR. To control the effect of the SNR, the deviation patterns of the time courses with similar SNR distribution were computed, and the observed deviation patterns for both BOLD signal increments and decrements were similar to the pattern for all selected voxels. This result suggested that the low SNR in the BOLD signal decrement was less likely to be the cause of the variations in deviation pattern. Nevertheless, the effect of SNR could not be completely excluded, and obtaining data with a higher SNR could increase the reliability of the results.

Apart from the BOLD signal decrement in the present study, previous studies also demonstrated another type of stimulus-induced BOLD signal decrease that was relative to the baseline condition, which was named as the negative BOLD responses (Fransson et al., 1999; Shmuel et al., 2002, 2006; Boorman et al., 2010; Gonzalez-Castillo et al., 2012; Klingner et al., 2015). For example, visual stimuli could elicit negative BOLD responses around the classical positive BOLD responses in the human visual cortex (Shmuel et al., 2002). Testing the linearity presumptions under the experimental settings for negative BOLD responses will help to reach a more general conclusion about the linearity of signal decrements in the future. In addition, dividing the BOLD responses into BOLD signal increments and decrements only focuses on one aspect of the temporal dynamics of BOLD responses. The BOLD responses demonstrate widely diverging temporal signatures across brain regions (Gonzalez-Castillo et al., 2012), thus further explorations are invited to examine the linearity presumption beyond the scope of BOLD signal increments and decrements.

## Conclusion

In summary, the present study examined the linearity of BOLD signal increments and decrements at a voxel-wise level. Unlike the uniform overestimation pattern in the BOLD signal increments, the BOLD signal decrements to a transient stimulus (e.g., 1-s stimulus) showed patterns varying from underestimation to overestimation. Further studies are required to gain more insight into potential physiological causes.

## Data Availability Statement

The data and stimulus code have been made available in an online repository: https://osf.io/w78a4/.

## Ethics Statement

The studies involving human participants were reviewed and approved by the Institutional Review Board of the Department of Psychology and the Center for Biomedical Imaging Research in Tsinghua University. The participants provided their written informed consent to participate in this study.

## Author Contributions

YL and XZ data were collected and analyzed. YL, XZ, and PS prepared the first draft of the manuscript. All authors conceived and designed the study and contributed to the final version of the manuscript.

## Conflict of Interest

The authors declare that the research was conducted in the absence of any commercial or financial relationships that could be construed as a potential conflict of interest.

## Publisher’s Note

All claims expressed in this article are solely those of the authors and do not necessarily represent those of their affiliated organizations, or those of the publisher, the editors and the reviewers. Any product that may be evaluated in this article, or claim that may be made by its manufacturer, is not guaranteed or endorsed by the publisher.

## Footnotes

1
http://afni.nimh.nih.gov/afni/

2
http://gru.stanford.edu/doku.php/mrTools/tutorialsRetinotopy

3
http://www.freesurfer.net/

4
http://gru.stanford.edu/doku.php/mrTools/tutorialsEventRelated
