# Supplementary material for: Voxel-Wise Linearity Analysis of Increments and Decrements in BOLD Responses in Human Visual Cortex Using a Contrast Adaptation Paradigm
Source: Front Hum Neurosci. 2021 Aug 31;15:541314. doi: 10.3389/fnhum.2021.541314 (PMC8439421; doi:10.3389/fnhum.2021.541314)
Supplement: Supplementary file 1 [file Data_Sheet_1.docx]

**Supplementary Material for “Voxel-wise Linearity Analysis of Increments and Decrements in BOLD Responses in Human Visual Cortex Using a Contrast Adaptation Paradigm”**

**Yun Lin^1^, Xi Zhou^1^, Yuji Naya^2^, Justin L. Gardner^3^, Pei Sun^1, 4, 5*^**

^1^Department of Psychology, School of Social Sciences, Tsinghua University, Beijing, China

^2^School of Psychological and Cognitive Sciences, Peking University, Beijing, China

^3^Department of Psychology, Stanford University, Stanford, CA, USA

^4^Tsinghua Laboratory of Brain and Intelligence, Tsinghua University, Beijing, China

^5^Laboratory for Cognitive Brain Mapping, RIKEN Center for Brain Sciences, Wako, Japan

*** Correspondence:**Pei Sun
[peisun@tsinghua.edu.cn](mailto:peisun@tsinghua.edu.cn)

**Generating randomized distribution using simulated time courses with temporal autocorrelation.** To verify the reliability of the result of the activated voxels selection, the false positive rates for the selected voxels were calculated based on a randomized distribution using simulated time courses with temporal autocorrelation. To generate the simulated time courses, the time course of the visual stimuli was shuffled and was convoluted with a canonical two-gamma HRF. The formula for the two-gamma HRF is given below (the same as **Equation (2)** in the main text):

$$h\left( t \right)=\left\{ \begin{aligned} A\left( \frac{\left( t-t_{\mathrm{onset}} \right)^{\alpha_{1}-1}{\beta_{1}}^{\alpha_{1}}e^{-\beta_{1}\left( t-t_{\mathrm{onset}} \right)}}{\Gamma\left( \alpha_{1} \right)}-c\frac{\left( t-t_{\mathrm{onset}} \right)^{\alpha_{2}-1}{\beta_{2}}^{\alpha_{2}}e^{-\beta_{2}\left( t-t_{\mathrm{onset}} \right)}}{\Gamma\left( \alpha_{2} \right)} \right), t\geq t_{\mathrm{onset}} \\ 0, t<t_{\mathrm{onset}} \end{aligned} \right.$$

The parameters that were used in Statistical Parametric Mapping (SPM) were applied to form HRF (Lindquist, Loh, Atlas, & Wager, 2009): A = 1, α_1_ = 6, α_2_ = 16, β_1_ = β_2_ = 1, c = 1/6, t_onset_ = 0. A total of 100000 simulated time courses were created, and the values of r^2^ for these time courses were used to form the randomized distribution for r^2^. The threshold of r^2^ with a false positive rate of 0.001 was 0.51, which was higher than that for the randomized distribution mentioned in the main text (≈ 0.47). The values of r^2^ for all selected voxels were higher than 0.51. Then the false positive rates for the voxels in V1 were corrected using the false discovery rate (FDR) approach (Benjamini & Hochberg, 1995). The corrected false positive rates were smaller than 0.005 for all selected voxels. These results suggested that the selected voxels were activated by the visual stimuli in the localizer experiment.

**References**

Benjamini, Y., & Hochberg, Y. (1995). Controlling the False Discovery Rate - a Practical and Powerful Approach to Multiple Testing. *Journal of the Royal Statistical Society Series B-Statistical Methodology, 57*(1), 289-300. doi:10.1111/j.2517-6161.1995.tb02031.x

Lindquist, M. A., Loh, J. M., Atlas, L. Y., & Wager, T. D. (2009). Modeling the hemodynamic response function in fMRI: Efficiency, bias and mis-modeling. *Neuroimage, 45*(1), S187-S198. doi:10.1016/j.neuroimage.2008.10.065

**Supplementary Table 1.** Results of Wilcoxon signed ranks tests comparing the values of the Dice index across BOLD signal increments and decrements. The values of the Dice index for BOLD signal increments were higher than that for BOLD signal decrements for all stimulus durations.

| Stimulus Duration | *Z* | *p* | adjusted-*p* |
| --- | --- | --- | --- |
| Measured: 3 s; Prediction from: 1 s | 12.039 | <0.001 | <0.001 |
| Measured: 6 s; Prediction from: 1 s | 10.436 | <0.001 | <0.001 |
| Measured: 6 s; Prediction from: 3 s | 7.487 | <0.001 | <0.001 |

Note: Adjusted-*p* values were calculated to correct the multiple comparisons problem. Adjusted-*p* = 1 – ( 1 – *p* )^3^.

**Supplementary Table 2.** Results of Wilcoxon signed ranks tests comparing the values of the Dice index across stimulus durations. For both BOLD signal increments and decrements, the values of the Dice index were higher when the responses to the 3-s stimuli were used to predict the measured responses comparing with when the responses to the 1-s stimuli were used.

| Response Type | Stimulus Duration | *Z* | *p* | adjusted-*p* |
| --- | --- | --- | --- | --- |
| BOLD Signal Increments | Measured: 3 s; Prediction from: 1 s  vs.  Measured: 6 s; Prediction from: 1 s | 5.394 | <0.001 | <0.001 |
|  | Measured: 3 s; Prediction from: 1 s  vs.  Measured: 6 s; Prediction from: 3 s | -11.589 | <0.001 | <0.001 |
|  | Measured: 6 s; Prediction from: 1 s  vs.  Measured: 6 s; Prediction from: 3 s | -12.479 | <0.001 | <0.001 |
| BOLD Signal Decrements | Measured: 3 s; Prediction from: 1 s  vs.  Measured: 6 s; Prediction from: 1 s | -0.425 | 0.671 | 0.999 |
|  | Measured: 3 s; Prediction from: 1 s  vs.  Measured: 6 s; Prediction from: 3 s | -12.238 | <0.001 | <0.001 |
|  | Measured: 6 s; Prediction from: 1 s  vs.  Measured: 6 s; Prediction from: 3 s | -12.155 | <0.001 | <0.001 |

Note: Adjusted-*p* values were calculated to correct the multiple comparisons problem. Adjusted-*p* = 1 – ( 1 – *p* )^6^.

**Supplementary Table 3.** The percentages of the voxels with values of the Dice index higher than the thresholds for the chance level (false positive rate < 0.05, one-tailed, Bonferroni corrected).

| Response Type | Stimulus Duration | Percentage of voxels  above thresholds |
| --- | --- | --- |
| BOLD Signal Increments | Measured: 3 s; Prediction from: 1 s | 65.5% |
|  | Measured: 6 s; Prediction from: 1 s | 62.8% |
|  | Measured: 6 s; Prediction from: 3 s | 84.8% |
| BOLD Signal Decrements | Measured: 3 s; Prediction from: 1 s | 12.6% |
|  | Measured: 6 s; Prediction from: 1 s | 26.0% |
|  | Measured: 6 s; Prediction from: 3 s | 58.7% |

**Supplementary Table 4.** Results of Kolmogorov-Smirnov one-sample tests comparing the distributions of the contrast index for the amplitude of HRFs with normal distributions with their mean equal to 0 and with the same standard deviations as the distributions for the contrast index.

| Response Type | Stimulus Duration | *D* | *p* | adjusted-*p* |
| --- | --- | --- | --- | --- |
| BOLD Signal Increments | Measured: 3 s;  Prediction from: 1 s | 0.601 | <0.001 | <0.001 |
|  | Measured: 6 s;  Prediction from: 1 s | 0.749 | <0.001 | <0.001 |
|  | Measured: 6 s;  Prediction from: 3 s | 0.347 | <0.001 | <0.001 |
| BOLD Signal Decrements | Measured: 3 s;  Prediction from: 1 s | 0.048 | 0.665 | 0.999 |
|  | Measured: 6 s;  Prediction from: 1 s | 0.174 | <0.001 | <0.001 |
|  | Measured: 6 s;  Prediction from: 3 s | 0.292 | <0.001 | <0.001 |

Note: Adjusted-*p* values were calculated to correct the multiple comparisons problem. Adjusted-*p* = 1 – ( 1 – *p* )^6^.

**Supplementary Table 5.** Descriptive statistics for the distributions of the contrast index for the amplitude of HRFs. The contrast index is close to 0 when the linear prediction and the measured response have similar amplitudes, larger than 0 when the linear prediction overestimates the measured response, and smaller than 0 when the underestimation exists. The column “overestimation (%)” indicates the percentage of voxels with their contrast index above 0.

| Response Type | Stimulus Duration | Mean | Median | Standard Deviation | Overestimation (%) |
| --- | --- | --- | --- | --- | --- |
| BOLD Signal Increment | Measured: 3 s; Prediction from: 1 s | 0.266 | 0.299 | 0.175 | 93.7% |
|  | Measured: 6 s; Prediction from: 1 s | 0.386 | 0.421 | 0.171 | 97.8% |
|  | Measured: 6 s; Prediction from: 3 s | 0.136 | 0.128 | 0.175 | 83.0% |
| BOLD Signal Decrement | Measured: 3 s; Prediction from: 1 s | 0.012 | 0.012 | 0.348 | 52.5% |
|  | Measured: 6 s; Prediction from: 1 s | -0.128 | -0.113 | 0.342 | 34.5% |
|  | Measured: 6 s; Prediction from: 3 s | -0.152 | -0.163 | 0.261 | 22.4% |

**Supplementary Table 6.** The comparison of deviation patterns across the low SNR group (n = 111) and the high SNR group (n = 112). The “overestimation (%)” indicates the percentage of voxels with their contrast index above 0.

| Response Type | Stimulus Duration | Overestimation (%) for low SNR group | Overestimation (%) for high SNR group |
| --- | --- | --- | --- |
| BOLD Signal Increment | Measured: 3 s; Prediction from: 1 s | 90.1% | 97.3% |
|  | Measured: 6 s; Prediction from: 1 s | 95.5% | 100.0% |
|  | Measured: 6 s; Prediction from: 3 s | 79.3% | 86.6% |
| BOLD Signal Decrement | Measured: 3 s; Prediction from: 1 s | 57.7% | 47.3% |
|  | Measured: 6 s; Prediction from: 1 s | 36.0% | 33.0% |
|  | Measured: 6 s; Prediction from: 3 s | 27.0% | 17.9% |

**Supplementary Table 7.** Results for Kolmogorov–Smirnov tests assessing the differences in the distributions of the contrast index between the low SNR group (n = 111) and the high SNR group (n = 112).

| Response Type | Stimulus Duration | *D* | *p* | adjusted-*p* |
| --- | --- | --- | --- | --- |
| BOLD Signal Increment | Measured: 3 s; Prediction from: 1 s | 0.181 | 0.045 | 0.243 |
|  | Measured: 6 s; Prediction from: 1 s | 0.146 | 0.172 | 0.678 |
|  | Measured: 6 s; Prediction from: 3 s | 0.173 | 0.064 | 0.327 |
| BOLD Signal Decrement | Measured: 3 s; Prediction from: 1 s | 0.111 | 0.480 | 0.980 |
|  | Measured: 6 s; Prediction from: 1 s | 0.100 | 0.607 | 0.996 |
|  | Measured: 6 s; Prediction from: 3 s | 0.184 | 0.041 | 0.221 |

Note: Adjusted-*p* values were calculated to correct the multiple comparisons problem. Adjusted-*p* = 1 – ( 1 – *p* )^6^.

**Supplementary Figure 1.** Simulated BOLD time courses and their frequency distributions. (**a**) The simulated BOLD time course for the localizer experiment. The black dashed line is a demonstration of the boxcar functions modeling the time course of the visual stimuli. (**c**) The simulated BOLD time course for a single run (91-890 volume) in the contrast adaptation experiment. Results showed that changing the order of the visual stimuli did not affect the overall frequency distribution. (**e**) The simulated recombined time course for the BOLD signal increments to 6-s stimuli. (**b**), (**d**), and (**f**) are the frequency distributions for the time courses presented in (**a**), (**c**), and (**e**). The black dashed line in (**f**) indicates the frequency corresponding to 10 cycles per time course, which is the high-pass frequency for the recombined time courses.

To determine the cutoffs for temporal filtering, the frequency distributions for the simulated BOLD time courses were calculated. The simulated time courses were constructed by convolving the time courses of the visual stimuli with a canonical two-gamma hemodynamic response function (HRF). The time courses of the visual stimuli were modeled as boxcar functions (for instance, the black dashed line in **(a)**). The formula for the two-gamma HRF is given below (this formula is the same as **Equation (2)** in the main text):

$$h\left( t \right)=\left\{ \begin{aligned} A\left( \frac{\left( t-t_{\mathrm{onset}} \right)^{\alpha_{1}-1}{\beta_{1}}^{\alpha_{1}}e^{-\beta_{1}\left( t-t_{\mathrm{onset}} \right)}}{\Gamma\left( \alpha_{1} \right)}-c\frac{\left( t-t_{\mathrm{onset}} \right)^{\alpha_{2}-1}{\beta_{2}}^{\alpha_{2}}e^{-\beta_{2}\left( t-t_{\mathrm{onset}} \right)}}{\Gamma\left( \alpha_{2} \right)} \right), t\geq t_{\mathrm{onset}} \\ 0, t<t_{\mathrm{onset}} \end{aligned} \right.$$

The parameters that were used in Statistical Parametric Mapping (SPM) were applied to form HRF (Lindquist et al., 2009): A = 1, α_1_ = 6, α_2_ = 16, β_1_ = β_2_ = 1, c = 1/6, t_onset_ = 0. Applying other parameters for the HRF also did not affect the overall frequency distribution for the simulated time courses. After constructing the simulated time courses, the magnitudes of the BOLD signal at each frequency were obtained using the fast Fourier transform. The magnitude of the DC-frequency was not presented in the figures as it does not affect the selection of the parameters for temporal filtering.

**Reference**

Lindquist, M. A., Loh, J. M., Atlas, L. Y., & Wager, T. D. (2009). Modeling the hemodynamic response function in fMRI: Efficiency, bias and mis-modeling. *Neuroimage, 45*(1), S187-S198. doi:10.1016/j.neuroimage.2008.10.065

**Supplementary Figure 2.** The voxels that had their false positive rate below 0.001 for one of the participants (see “Activated Voxels Selection” section for the calculation of r^2^). (**a**) The activation map for the localizer experiment. (**b**) The activation map for the contrast adaptation experiment.

**Supplementary Figure 3.** The mean measured BOLD responses and their linear predictions. (**a**) The mean measured BOLD responses for all selected voxels from all participants. The colors of the BOLD responses indicate the contrast level and the duration of the test stimuli. (**b**) The comparison between the mean measured BOLD responses and their linear predictions. The Dice index is a similarity index that ranges from -1 to 1, in which higher Dice index indicates higher similarity between measured BOLD response and its linear prediction.

**Supplementary Figure 4.** The distributions of the HRF amplitude ratio for different BOLD response types and stimulus durations. The HRF amplitude ratio is close to 1 when linear prediction and measured response have similar amplitudes and is larger (or smaller) than 1 when linear prediction overestimates (or underestimates) measured response. The vertical dashed lines indicate that the HRF amplitude ratio equals 1. Each data point refers to the HRF amplitude ratio of one selected voxel.

**Supplementary Figure 5.** The distributions of the contrast index for the BOLD signal increments in each participant. The contrast index is close to 0 when linear prediction and measured response have similar amplitudes and is larger (or smaller) than 0 when linear prediction overestimates (or underestimates) measured response. The vertical dashed lines indicate that the contrast index equals 0. Each data point refers to the contrast index of one selected voxel.

**Supplementary Figure 6.** The distributions of the contrast index for the BOLD signal decrements in each participant. The contrast index is close to 0 when linear prediction and measured response have similar amplitudes and is larger (or smaller) than 0 when linear prediction overestimates (or underestimates) measured response. The vertical dashed lines indicate that the contrast index equals 0. Each data point refers to the contrast index of one selected voxel.

**Supplementary Figure 7.** The distributions of the contrast index for different BOLD response types and stimulus durations using a more flexible HRF. The contrast index is close to 0 when linear prediction and measured response have similar amplitudes, and is larger (or smaller) than 0 when linear prediction overestimates (or underestimates) measured response. The vertical dashed lines indicate that the contrast index equals 0. Each data point refers to the contrast index of one selected voxel. Each distribution includes all selected voxels from all participants.

**Supplementary Figure 8.** The results of the linearity analysis for one participant after subtracting the fMRI signal of the last volume before each trial in the preprocessing stage. (**a**) The distributions of the Dice index for different BOLD response types and stimulus durations. The Dice index describes the similarity between measured BOLD response and its linear prediction. The vertical dashed lines indicate the upper thresholds for chance levels (false positive rate = 0.05, one-tailed, corrected), and having a Dice index above chance level suggests that the response to the short stimulus can predict response to the longer stimulus in a linear way. Each data point refers to the Dice index of one selected voxel. (**b**) The distributions of the contrast index for different BOLD response types and stimulus durations. The contrast index is close to 0 when linear prediction and measured response have similar amplitudes and is larger (or smaller) than 0 when linear prediction overestimates (or underestimates) measured response. The vertical dashed lines indicate that the contrast index equals 0. Each data point refers to the contrast index of one selected voxel.

**Supplementary Figure 9.** The eccentricity and SNR versus the amplitude of HRF in the contrast adaption experiment. (**a**) The eccentricity versus the amplitude of HRF in one participant. The slice number for the selected voxels was used as the parameter for eccentricity because the slices for the functional images were perpendicular to the calcarine sulcus and were extended from the occipital pole. The higher the slice number, the further the distance between the slice for the voxel and the occipital pole. (**b**) The SNR versus the amplitude of HRF for all selected voxels.


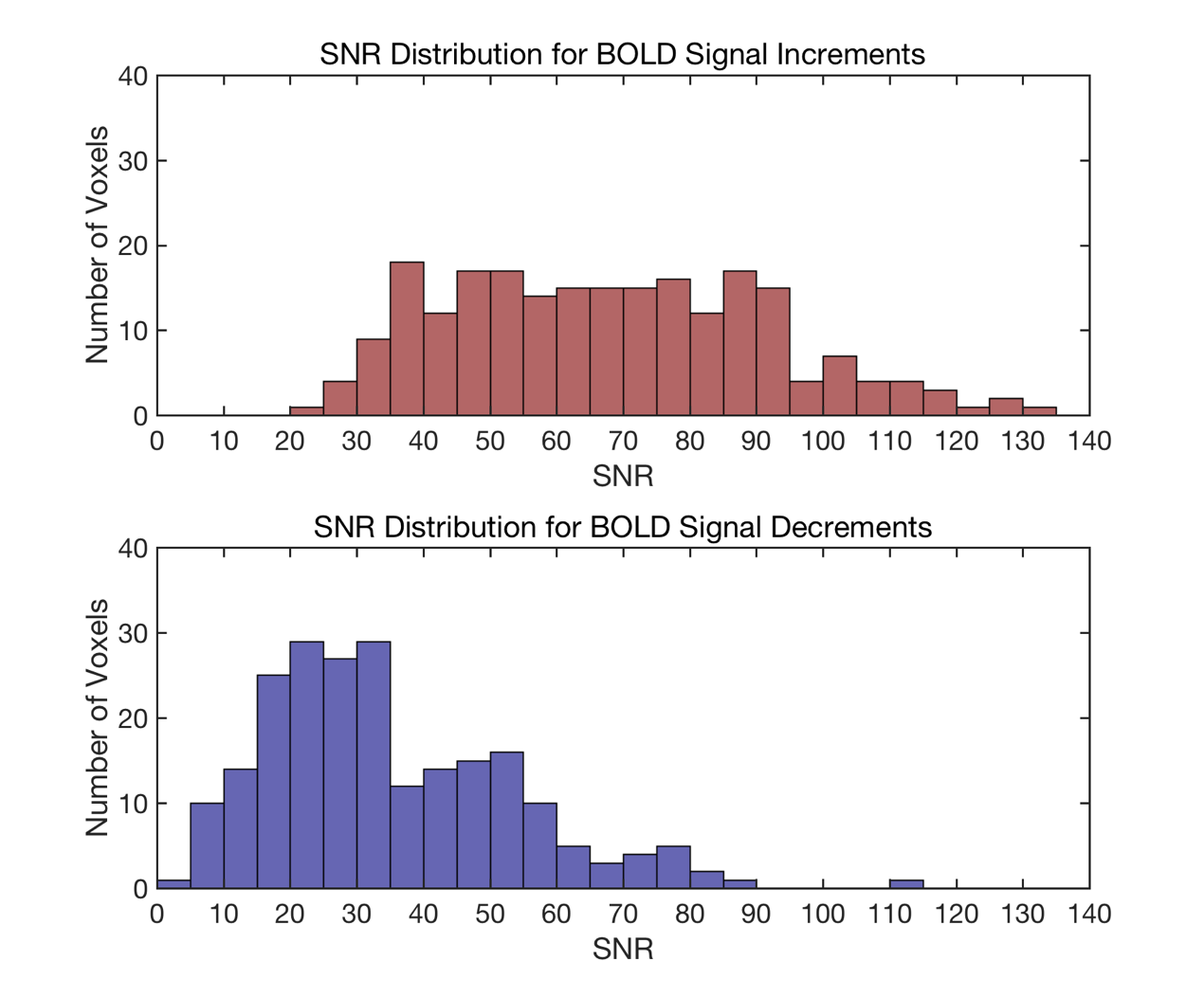
**Supplementary Figure 10.** The SNR distributions for the BOLD signal increments and decrements. Each data point refers to the SNR of one selected voxel.

**Supplementary Figure 11.** The distributions of the contrast index for the low SNR group and high SNR group using all frequencies except the task frequency as the noise band. The low SNR group (n = 111) and high SNR group (n = 112) were split by the median r^2^ of the corresponding BOLD response type. The vertical dashed lines indicate that the contrast index equals 0. Each data point refers to the contrast index of one selected voxel. Using other noise bands also yielded similar distributions.
